# Expertise-related functional brain network efficiency in healthy older adults

**DOI:** 10.1186/s12868-016-0324-1

**Published:** 2017-01-03

**Authors:** Julia C. Binder, Ladina Bezzola, Aurea I. S. Haueter, Carina Klein, Jürg Kühnis, Hansruedi Baetschmann, Lutz Jäncke

**Affiliations:** 1 Division of Gerontopsychology and Gerontology, Department of Psychology, University of Zurich, Zurich, Switzerland; 2 International Normal Aging and Plasticity Imaging Center (INAPIC), University of Zurich, Zurich, Switzerland; 3 University Research Priority Program (URPP) “Dynamics of Healthy Aging”, University of Zurich, Zurich, Switzerland; 4 Division of Neuropsychology, Department of Psychology, University of Zurich, Zurich, Switzerland; 5 Center for Integrative Human Physiology, University of Zurich, Zurich, Switzerland; 6 Center for Lifespan Psychology, Max Planck Institute for Human Development, Berlin, Germany

**Keywords:** Healthy aging, Expertise, Functional brain networks, Electroencephalography (EEG), Graph theory, Network-based statistics (NBS)

## Abstract

**Background:**

In view of age-related brain changes, identifying factors that are associated with healthy aging are of great interest. In the present study, we compared the functional brain network characteristics of three groups of healthy older participants aged 61–75 years who had a different cognitive and motor training history (multi-domain group: participants who had participated in a multi-domain training; visuomotor group: participants who had participated in a visuomotor training; control group: participants with no specific training history). The study’s basic idea was to examine whether these different training histories are associated with differences in behavioral performance as well as with task-related functional brain network characteristics. Based on a high-density electroencephalographic measurement one year after training, we calculated graph-theoretical measures representing the efficiency of functional brain networks.

**Results:**

Behaviorally, the multi-domain group performed significantly better than the visuomotor and the control groups on a multi-domain task including an inhibition domain, a visuomotor domain, and a spatial navigation domain. In terms of the functional brain network features, the multi-domain group showed significantly higher functional connectivity in a network encompassing visual, motor, executive, and memory-associated brain areas in the theta frequency band compared to the visuomotor group. These brain areas corresponded to the multi-domain task demands. Furthermore, mean connectivity of this network correlated positively with performance across both the multi-domain and the visuomotor group. In addition, the multi-domain group showed significantly enhanced processing efficiency reflected by a higher mean weighted node degree (strength) of the network as compared to the visuomotor group.

**Conclusions:**

Taken together, our study shows expertise-dependent differences in task-related functional brain networks. These network differences were evident even a year after the acquisition of the different expertise levels. Hence, the current findings can foster understanding of how expertise is positively associated with brain functioning during aging.

## Background

Normal aging is accompanied by structural and functional brain changes [1–4]. Insights into the neurobiological basis of cognitive aging are important to develop interventions and understand their underlying neurophysiological mechanisms. Intact cognitive functioning draws on an efficient interaction of several brain areas, each specialized for information processing in certain domains [5]. However, such large-scale structural and functional brain networks undergo changes in old age [6], and these changes parallel age-related cognitive decline [7, 8].

Functional brain networks are based on functional connectivity [e.g., 9]. Functional connectivity refers to statistical measures of brain activity between different brain regions [10]. Graph theoretical analyses of functional connectivity measures originating from different neurophysiological recording techniques (e.g., electroencephalography: EEG; magnetencephalography: MEG; and resting state functional magnetic resonance imaging: rs-fMRI) generally reveal networks characterized by a small-world topology in most human study samples [9, 11, 12]. Small-world topology consists of specialized information processing modules across which information is transferred and integrated efficiently [13].

Networks consist of nodes and edges; nodes represent brain regions, while edges represent the connections between the nodes. Networks can be based on anatomical or functional measures and can be either binary (an existing connection vs. no connection) or weighted by the strength of the connections. The two most important measures to characterize networks are the clustering coefficient and the characteristic path length [14]. The clustering coefficient refers to the local connectedness of nodes and is the fraction of the existing edges among the neighbors of a node in relation to the theoretically possible edges among these neighbors. Nodes with a high clustering coefficient are central information processing hubs in a network. The distance between two nodes is defined as the smallest number of edges that have to be traversed from one to another node. The mean of the distance of all pairs of nodes is referred to as the characteristic path length, a measure of how efficiently a network is connected. Small-world networks designate networks with a high clustering coefficient and a short characteristic path length [9, 11, 13, 15].

Resting state functional brain networks have shown age-related alterations [16]. A cross-sectional rs-fMRI study involving a life-span sample of 913 healthy participants aged 13–85 years revealed that age was associated with a decreased strength of functional connectivity density (number of connections) in the default mode network and the dorsal attention network [17]. Another study found that the modular structure of older adults’ resting state functional brain networks differed from young adults’ networks in module size and their interconnections. Modules in older adults were generally smaller and more local. Furthermore, the number of posterior-central module connections was higher, while numbers of posterior-frontal and central-frontal module connections were lower [18]. EEG studies found comparable differences in resting state activity. A cross-sectional study including young, middle-aged, and older adults found age-related modulations of functional brain network integration in the delta, theta, and upper alpha (alpha2) frequency bands [19]. More specifically, the characteristic path length of the network correlated positively with age in the delta and theta band, while there was a negative correlation in the upper alpha band. Furthermore, the characteristic path length in patients affected by Alzheimer’s disease was significantly longer in the theta [20] and beta band [21] than in healthy control participants. Task-related functional brain networks have been investigated less frequently. An fMRI study found that older adults showed less functional connectivity between frontal and parietal regions during task-switching as compared to younger controls [22].

The potential to increase and/or modify brain structure and function is preserved across the adult life span [23]. Certain lifestyle factors, such as social activities, cognitive stimulation, and exercise, are associated with better cognitive and brain functioning in old age [24]. To date, there are only a few findings of such experience-dependent associations with functional brain network characteristics. For example, resting state functional small-worldness was higher in healthy middle-aged adults who regularly practiced yoga or meditation than in matched controls [25]. Furthermore, the meditation and yoga groups performed higher on fluid intelligence measures, and fluid intelligence was positively correlated with mindfulness. Working memory training in a group of young adults showed training-induced increases in theta band small-world topology during resting state EEG [26]. With regard to healthy old age, the simultaneous multi-domain training of two different cognitive functions ameliorated performance on both the simultaneous and the separate training task conditions, while the sequential training only improved performance on each isolated task, but not on their simultaneous combination [27]. In line with these training-related performance improvements, EEG measurements revealed training-related increases in midline frontal theta power and long-range theta coherence between frontal and posterior brain regions only in the multi-domain training group [27]. Theta power and theta coherence between frontal and posterior brain regions have been associated with increased cognitive control [28–30].

In the present study, we used high-density EEG to investigate group differences in functional connectivity and functional small-world network characteristics of healthy older adults who had participated in two different, intense, and long-lasting types of cognitive training about one year prior to EEG recordings. Here, we examine whether the particular expertise acquired to master a practiced task is reflected in functional brain network features. Thus, we tested whether different expertise levels influence brain activation patterns even a year after the training. Studying expertise-related behavioral, neurophysiological, and neuroanatomical between-group differences cross-sectionally has a long tradition in plasticity and expertise research. For example, cross-sectional studies with musicians [31, 32], sportsmen [33–36], chess-players [37, 38], or with participants with different intellectual abilities [39] have been conducted in the past and present [40]. We applied the same research strategy here by comparing older participants with different cognitive training histories. We hypothesize that there are task-related functional brain network distinctions between the groups that differed with respect to the expertise they had acquired during training one year before. The multi-domain group trained visuomotor, inhibition, and spatial navigation skills simultaneously, while the visuomotor group trained visuomotor function only. Thus, we hypothesize that only the multi-domain group acquired expertise reflected in functional brain network efficiency involved in controlling visuomotor, spatial, and inhibitory functions (e.g., occipital, temporal, parietal, and frontal areas), while the single-domain group acquired expertise that involved functional brain networks controlling visuomotor functions (e.g., visual and motor areas). To investigate functional brain networks in the theta and alpha bands, we calculated instantaneous coherence as a measure of functional connectivity during multi-domain task performance to compare functional brain networks between groups; we also computed small-world indices (weighted node degree). Theta and alpha frequency bands have been associated with working memory and attention [41], and have shown to be modifiable by cognitive training [26, 27, 42].

## Methods

### Participants

We investigated three groups of participants differing in their expertise level due to their particular cognitive and motor training history. The first group of participants had undergone multi-domain training and the second group of participants had undergone visuomotor function training approximately a year before (the time interval between posttest and EEG session did not differ between groups [*t*(27) = −.76, *p* > .40]; both groups: *M* = 11.7 months, *SD* = 0.72, range 11–14 months). Both groups had been randomly assigned to the training groups and practiced the iPad-based Hotel Plastisse training at home during 50 training sessions over 10 weeks [43, 44] (except for one visuomotor participant who had only completed 42 training sessions). Each training session lasted about 45 min and consisted of 5 different training tasks. The third group of newly recruited participants (control group) did not have a particular training background, but was matched for age and gender. Furthermore, the control group did not differ from the other two groups with respect to important study sample characteristics (see Table 1). The study investigators were not blinded to the group assignment.

**Table 1:** Characteristics of the whole sample and of each group separately

Demographics	All	Multi-domain training	Visuomotor training	Control participants
Sample size (f, m)	46 (28, 18)	13 (9, 4)	16 (8, 8)	17 (11, 6)
Age	70.28 (2.87)	71.02 (2.57)	70.23 (2.18)	69.77 (3.62)
MMSE	29.02 (0.80)	29.00 (0.91)	29.06 (0.85)	29.00 (0.71)
Depression	1.22 (1.41)	1.46 (1.33)	1.13 (1.50)	1.12 (1.45)
Handedness	13.09 (2.73)	12.38 (0.96)	12.13 (0.50)	14.53 (4.06)
School education	9.97 (1.90)	9.96 (1.90)	9.97 (2.15)	9.97 (1.77)
Vocabulary	32.48 (2.05)	32.31 (2.43)	32.88 (1.71)	32.24 (2.11)

Means and standard deviations (in parentheses) are indicated. MMSE [Mini-Mental Status Examination; 45]; depression [Geriatric Depression Scale (GDS); 46, 47]; handedness [49]; vocabulary [MWT-B; 48]

#### Recruitment and study admission

Our study sample consisted of 46 healthy older participants aged 61–75. Participants in the original training study [44] were contacted by phone and asked whether they were willing to participate in an additional EEG study. The additional control participants were recruited based on the following criteria: age 61–75 years, fluent in German, self-reported right-handedness, neurologically and psychiatrically healthy, no severe manual motor deficiencies. The participants were tested with the Mini-Mental Status Examination [MMSE; 45] in order to exclude potential participants with cognitive impairment. All participants filled in an extensive health questionnaire and were screened for depressive symptoms with the Geriatric Depression Scale [GDS; 46, 47]. For participation in the EEG measurement and additional cognitive testing, participants were reimbursed 60 CHF (approximately 60 USD). The study was conducted according to the principles of the Declaration of Helsinki and was approved by the ethics committee of the Department of Psychology at the University of Zurich. All participants gave written informed consent prior to the study.

Fourteen of originally 21 multi-domain training participants and 16 of originally 21 visuomotor training participants took part in the present EEG study (one participant of the multi-domain group was excluded because of a cardiac pacemaker that strongly disturbed EEG data quality, hence the final group size was 13). Additionally, 24 participants were recruited as controls to participate in the testing session. We excluded seven of them (two yielded bad EEG data quality, four were excluded due to their cognitive status, and one participant took antidepressants).

#### Characteristics of the final study sample

Our study sample had a mean age of *M* = 70.28 years (*SD* = 2.87), did not show any cognitive impairments in the MMSE screening (*M* = 29.02, *SD* = .80, range 27–30 points), did not show depressive symptoms (*M* = 1.22, *SD* = 1.41), was right-handed (*M* = 13.09, *SD* = 2.73), had an average school education of *M* = 9.97 years (*SD* = 1.90), and showed a vocabulary score [48] indicating high average crystallized intelligence (*M* = 32.48, *SD* = 2.05). Details of the study characteristics for the whole sample and each of the three groups are shown in Table 1. The three groups did not differ with respect to the ratio of male to female participants (*χ* ^2^(2) = 1.28, *p* = .527), age [*F*(2, 43) = .691, *p* = .507], MMSE [*F*(2,43) = .030, *p* = .970], depressive symptoms [GDS; *F*(2, 43) = .262, *p* = .771], years of school education [*F*(2, 43) = .000, *p* = 1.00], or vocabulary knowledge [*F*(2,43) = .452, *p* = .639]. Two control participants indicated ambidexterity [49], hence handedness differed significantly between groups [*F*(2, 43) = 4.360, *p* = .019].

### Training history

While the naive control group did not have a particular cognitive or motor training history and was newly recruited, the multi-domain and visuomotor group had participated in a training prior to the present study.

#### Original multi-domain training

The participants had trained five different multi-domain tasks [43] approximately a year before. All five training tasks were designed similarly: they required the participants to simultaneously handle a spatial navigation task, an inhibition task, and a visuomotor function task. Participants first had to memorize a labyrinth. Subsequently, they were walked through the memorized, virtual labyrinth. At every crossroad, they had to decide on the correct direction (recall of the labyrinth; spatial navigation task). Between two crossroads, participants had to aim at targets as precisely as possible (visuomotor task) and inhibit their reaction to no-go stimuli (inhibition task). The training was adaptive according to the participants’ performance across the 50 training sessions.

#### Original visuomotor training

The visuomotor function training completed approximately a year before consisted of five training tasks to practice eye-hand coordination [43]. These tasks were designed to train unimanual or bimanual hand or finger movements by aiming at targets as precisely as possible. The training was adaptive according the participants’ performance across the 50 training sessions.

### Procedure

The EEG measurement began with an EEG resting state acquisition. Participants subsequently completed different tasks of the Hotel Plastisse training software on an iPad [43]. At the end, the resting state measurement was repeated. In order to prevent sequence effects, the iPad tasks were presented in two different orders, and the type of order was counterbalanced across the multi-domain and the control groups, while the visuomotor group had a different order involving an additional visuomotor task. In the present study, only the EEG measurement during the multi-domain task (called wine tasting) is of interest.

#### Multi-domain task

All participants performed the wine tasting task, one of the five multi-domain tasks of the original multi-domain training. This task required participants to simultaneously handle a spatial navigation task (spatial navigation domain), an inhibition task (inhibition domain), and a visuomotor task (visuomotor domain; in total three domains). Participants were first presented with a 3D-video of a labyrinth consisting of seven crossroads. The direction at the crossroads had to be memorized. During retrieval, participants were walked through the same labyrinth again. At every crossroad, they had to decide on the correct direction (spatial navigation domain). Between two crossroads, participants were presented with a continuous stream of go and no-go stimuli (inhibition domain, 288 go stimuli, 96 no-go stimuli, delay between two stimuli was 0.94 s). They had to react to new wine bottles (go stimuli), while broken ones were to be ignored (no-go stimuli). In addition, the new wine bottles had to be hit as precisely as possible (visuomotor domain). The task consisted of two different labyrinths that were presented subsequently. Each of them again involved seven crossroads to remember. The task duration was about 6–10 min with some inter-individual variability since participants self-paced the start of retrieval and the start of the second labyrinth. The dependent variable was the mean percentage of correct performance on all three tasks carried out simultaneously [actually correct reactions of all three domains divided by all possible correct reactions of all three domains; 43].

The three participant groups had gained different expertise levels through the training carried out approximately one year before. It is important to note that the multi-domain group had originally practiced the multi-domain task, and the visuomotor group had practiced visuomotor tasks and did not have any experience with the multi-domain task. However, prior visuomotor training could be beneficial to the visuomotor domain of the current multi-domain task. The control group was completely naive as they had never been exposed to the Hotel Plastisse training before, but they were similar with respect to their demographics (cf. Table 1). Considering the different levels of participants’ expertise, we fixed task difficulty to an intermediate level (level 28) that was demanding for the participants with a training history and still manageable for the naive controls. For technical reasons, two participants only performed on one labyrinth, one of whom performed on a higher level with eight instead of seven crossroads. However, the performance score does not depend on the number of traversed labyrinths as it is calculated as the percentage of actually correct out of potentially correct reactions. The performance of the participant with a higher difficulty level did not influence the overall behavioral results, as the findings did not change when that person was excluded from the analyses.

### Analyses of behavioral data

We compared performance (percentage correct) between groups (multi-domain, visuomotor, control group) with the Kruskal–Wallis test (non-parametric equivalence to a one-way ANOVA, according to unequal variances across groups). To investigate pairwise group comparisons, we computed post hoc Dunn–Bonferroni tests. The criterion of statistical significance was a *p* value of *p* < .05 (*p* values represent asymptotic significances). We calculated *r* as a measure of effect size by dividing the *z* value of the Dunn–Bonferroni tests by the square root of the number of participants, and taking the absolute value of this quotient.‬ An effect size of *r* = .1 was considered as small, *r* = .3 as medium, and *r* = .5 as large [50]. We performed these analyses with SPSS 22 (SPSS Inc, Chicago, IL, USA; http://www-01.ibm.com/software/analytics/spss/).

### Electroencephalographic (EEG) recording and raw data processing

Participants were seated in a sound-shielded Faraday cage. We instructed them to sit comfortably and remain as relaxed as possible during the whole EEG measurement. We acquired high-density EEG with a 256-channel EEG Geodesic Netamps system (Electrical Geodesics, Eugene, OR, USA; www.egi.com). EEG recording was continuously sampled at 500 Hz with a low-pass filter of 100 Hz and a notch filter at 50 Hz. Cz served as the recording reference (vertex of head). Impedances were controlled after every second task and kept below 30 kOhm.

We preprocessed the raw EEG data in BrainVision Analyzer 2.0 (Brain Products GmbH, Munich, Germany; www.brainproducts.com). First, we excluded the channels of the outermost circumference (chin, neck) to a standard 204 electrode array [26]. We then filtered data off-line from 1 to 100 Hz including a notch filter at 50 Hz. To remove artifacts (heart beats, eye movements and blinks, muscular artifacts), we computed an independent component analysis [51]. Next, we topographically interpolated bad channels and ran the automated raw data inspection implemented in BrainVision Analyzer. We subsequently inspected data visually. The artifact-free data was re-referenced to the average reference and segmented into epochs of 2 s. Due to incompatibility between the iPad and the EEG system, we analyzed the data continuously (2 s epochs) and not based on events. The number of segments of the multi-domain task ranged from 128 to 276 due to individual differences in task duration and EEG data quality. These data provided the basis for the topographic scalp map and the functional brain network analyses.

### Topographic scalp map analyses

We exported the data from BrainVision Analyzer (generic data export) and calculated the power spectra of the frequency bands theta (6.5–8 Hz), alpha1 (8.5–10 Hz), and alpha2 (10.5–12 Hz) for each electrode averaged across all data segments per participant [26, 52, 53] using an in-house programmed Matlab script (Matlab R2015a, Mathworks Inc., MA, USA; www.mathworks.com). We chose these three frequency bands a priori due to their importance in memory and attentional processes [29, 41]. We then averaged the power spectra for six electrode clusters, each consisting of 28 electrodes [26, 53]: three anterior clusters (right, middle, left) and three posterior clusters (right, middle, left; see Appendix Table 5 and Fig. 3 for details). For the analyses of the topographic scalp map data, we performed Kruskal–Wallis tests according to deviations from a normal distribution (non-parametric equivalence to a one-way ANOVA) for each of the three frequency bands and each of the six electrode clusters with the factor group (multi-domain, visuomotor, control group). The criterion of statistical significance was a *p* value of *p* < .05 (asymptotic significance). We corrected for multiple comparisons by Bonferroni (*p* value divided by the number of tests: 0.05/18). Two participants showed power values beyond four standard deviations of the mean (one participant in the visuomotor group, one participant in the control group). Excluding these participants did not change the results. Hence, we report the analyses with all participants. We performed these analyses with SPSS 22 (SPSS Inc, Chicago, IL, USA; http://www-01.ibm.com/software/analytics/spss/).

### Connectivity analyses in intracranial space

The preprocessed and artifact-free 2 s segments were imported into the sLORETA toolbox for connectivity analyses [http://www.uzh.ch/keyinst/loreta; 54]. We calculated intracranial instantaneous coherence measures [26] across the centroid voxels between all 84 Brodmann areas (BA) as specified in the sLORETA toolbox (42 in each hemisphere) separately for the frequency bands of interest (theta, alpha1, alpha2). Analyzing graph-theoretical networks in intracranial space rather than on the basis of surface activity circumvents the problem of high correlations between neighboring electrodes [55] and has been acknowledged as a methodological improvement [cf. 26]. In sLORETA, a standard head model using an MNI152 template is implemented [54]. The BAs were based on this standard model for all particpants, the centroid voxels of the BAs were pre-defined in the sLORETA toolbox. Instantaneous coherence has been used previously as a connectivity measure in functional brain network analyses [e.g., 26] and ranges from 0 (independent time series) to 1 [perfectly synchronous time series; for mathematical details, see 56].

#### Network-based statistics

The 84 × 84 connectivity matrices (according to the number of BAs from sLORETA) of each participant were then subjected to the Matlab toolbox NORNA (non-random network analyses), an extension of Network-based Statistics [NBS, https://www.nitrc.org/projects/nbs/; 57], to evaluate group differences in functional brain networks separately for the three frequency bands theta, alpha1, and alpha2. In a first step, we performed an *F*-test to evaluate group differences for each frequency band. Follow-up *t*-tests were computed to describe significant main effects of group more precisely. NBS (and its extension NORNA) is a non-parametric statistical method and tests networks as a whole, which is in contrast to traditional statistical network evaluations that perform a hypothesis test for each connection and correct the *p* values with a correction method for multiple testing. In this way, NBS accounts for dependencies between the connection values of an individual. In a first step, a *F*-test/*t*-test is calculated for each connection. Thus, edges exceeding an arbitrarily chosen *t*-threshold form a so-called supra-threshold network, constituting a subnetwork (or many disjoint subnetworks) of the network to be tested. The size of the biggest network, the so-called biggest component, serves as a test statistic. A simulation of the unknown distribution of the test statistic is performed by randomly permuting (5000 randomizations) the residuals after fitting a linear model for each connection between the participants. Thus, the within-subject dependencies of the connection values are preserved. The sizes of the biggest components constitute the simulated distribution. Hence, the null-hypothesis for a selected alpha error is then tested by comparing the size of the biggest component of the full network against the simulated distribution of the size of the biggest components of the simulated networks. NORNA is an extension of NBS that helps to find such an approximately minimal biggest component near the phase transition of a random network in a semi-automated, informed search. In addition, it provides intermediate and final results, and graphics (in the following we refer to the analyses as “NBS analyses”). The procedure mimics a family-wise error rate correction (FWE) of the traditional procedure, where a separate hypothesis test is performed for each edge. Irrespective of the threshold chosen, an FWE correction is guaranteed. The threshold affects the extent of a network. Since the connectivity values differ between frequency bands, the thresholds have to be chosen for each frequency band individually. Only the *F*-test in the theta band revealed a significant functional brain network for a main effect of group (3 components, thereof 1 significant: *t* = 5.6, *p* = .047, FWE-corrected, 49 nodes, 114 edges). Following the significant functional brain network difference in the theta band (*F*-test), we report pairwise comparisons between groups based on our hypotheses [a significant functional brain network was found for the *t*-test comparing the multi-domain vs. visuomotor group (2 components, thereof 1 significant: *t* = 3.6, *p* = .006, FWE-corrected, 18 nodes, 20 edges)]. Then, we took the Jülich Histological, the Harvard Oxford, and the Talairach Atlases implemented in FSL (http://fsl.fmrib.ox.ac.uk/fsl/fslwiki/Atlases) to describe the underlying BAs (centroid voxels) of the nodes of the functional brain network in more detail (see Table 2 for MNI coordinates of centroid voxels of the respective BAs reported in the results section). Spearman’s correlations (*r*_s_, two-tailed) were calculated to investigate how connectivity of the whole functional brain network and of individual edges related to performance. We show these correlations to give a clearer picture of the functional brain network revealed by the NBS analyses and therefore report them uncorrected for multiple comparisons. One participant in the multi-domain group showed values beyond 4 standard deviations of the mean for two edges. Excluding this participant for these particular edges did not change the correlations substantially. Consequently, we report the correlations for the whole sample. We visualized the functional brain networks with the BrainNet Viewer [http://www.nitrc.org/projects/bnv/; 58].

**Table 2:** Specification of the centroid voxels relevant for the NBS results

L/R	BA	MNI coordinates of centroid voxels from sLORETA		
		x	y	z
L	2	−45	−30	45
L	4a*	−35	−25	55
L	4b*	−35	−20	50
L	5	−15	−45	60
L	13	−40	−10	10
L	17a*	−10	−90	0
L	17b*	−15	−85	0
L	20	−45	−20	−30
L	36	−30	−30	−25
L	37	−45	−55	−15
L	40	−50	−40	40
L	42	−60	−10	15
R	5	15	−45	60
R	7	15	−65	50
R	28	20	−10	−25
R	34	15	0	−20
R	35	25	−25	−20
R	45	50	20	15

L, left hemisphere; R, right hemisphere; BA, Brodmann area derived from sLORETA. According to sLORETA, there are two centroid voxels in left BA 4 and left BA 17 (marked with asterisks). We therefore use the letters a and b to distinguish them in the results

#### Graph-theoretical small-worldness and regional node analyses

To characterize the functional brain network in the theta band differing between the multi-domain and the visuomotor group as revealed by the NBS analyses in more detail, we additionally calculated the graph-theoretical index weighted node degree centrality (sometimes termed strength; in the following referred to as weighted node degree). As a follow-up analyses, it was restricted to the multi-domain and the visuomotor groups.

In a first step, small-worldness was calculated. Small-world organization is a network characteristic implying high segregation and integration [59]. Functional network segregation refers to the presence of highly specialized modules with dense interconnections for specialized information processing (quantified by the clustering coefficient of a network), while functional integration refers to the efficient combination of these specialized information processes from distributed modules (quantified by the characteristic path length of a network). Typically, small-worldness is defined by the clustering coefficient *C* of the real data being higher than one of a random network (γ = C real/C random, γ ≫ 1), while the characteristic path length *L* of real data is short and comparable to a random network (λ = L real/L random, λ ~ 1). Mathematically, small-worldness (ϭ) is then defined as the ratio of gamma and lambda (ϭ = λ/γ) being >1. To evaluate small-worldness, absolute thresholds in the range from *r* = .65 to *r* = .95 in increments of 0.05 were applied to the mean connectivity matrix (instantaneous coherence) across all participants [26, 60]. For each threshold, network parameters were calculated with the Matlab-based Brain Connectivity Toolbox [www.brain-connectivity-toolbox.net; 59]. The random network was based on 100 randomizations. We applied the threshold for which sigma was the highest (ϭ = 1.08 in the theta frequency band for *r* = .95) on each individual connectivity matrix before computing further analyses (weighted node degree).

In a second step, we calculated weighted node degree to investigate efficiency in information processing within the functional brain network in the theta band differing between the multi-domain and the visuomotor group during multi-domain task performance as revealed by the NBS analyses. The degree of a node is defined as the number of edges connected to a particular node [59]. Weighted node degree takes into account the strength of these connections (e.g., the coherence values) by calculating the sum of weights of all edges of a node [61]. The mean of the weighted node degree of all nodes of the obtained functional brain network and the weighted node degree of each individual node of the obtained functional brain network was compared between the groups using Mann–Whitney *U* tests for independent samples (according to deviations from a normal distribution; exact significance is reported). We calculated *r* as a measure of effect size (in an analogous manner to the Kruskal–Wallis test, see above “Analyses of behavioral data” section). In order to investigate the relevance of weighted node degree for performance, we correlated mean weighted node degree of the whole functional brain network differing between the two groups as well as the weighted node degree of each individual node with performance (Spearman’s correlations, *r*_s_, two-tailed). These follow-up analyses are uncorrected for multiple comparisons as we intend to explore the functional brain network revealed by the NBS analyses more closely. One participant in the multi-domain group showed a value beyond four standard deviations of the mean for the weighted degree values of three nodes. Excluding this participant for these particular nodes did not change the result except for one correlation between weighted node degree and performance across both groups. For this particular correlation, we report the results with and without this participant. For all other analyses, we report the analyses for the whole sample.

## Results

Our main interest is focused on the comparison of the three groups that differed in their particular expertise with respect to differences in power spectra and functional brain network characteristics of the theta and alpha band frequencies.

### Performance on the multi-domain task

**Fig. 1:**
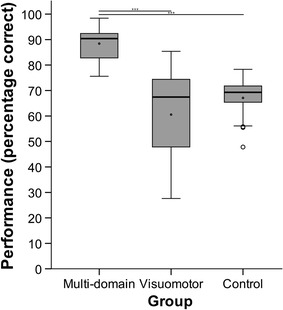
Percentage of performance in the multi-domain task (wine tasting) during EEG acquisition for the multi-domain group, the visuomotor group, and the control group. *Boxplots* show median (*black line*), mean (*black dot* in the interquartile range), *lower* and *upper* quartile (*box*), values between 1.5 and 3 times the interquartile range (*circles; 3 participants*). ***p < .001

The three groups’ performance (percentage correct) on the multi-domain task during the EEG measurement differed significantly (Kruskal–Wallis test with the factor group: *χ* ^2^(2) = 24.27, *p* < .001, see Fig. 1). Post-hoc Dunn–Bonferroni tests revealed that performance was significantly higher in the multi-domain group (performance *M* = 88.42, *SD* = 7.35, *Mdn* = 90.40) compared to the visuomotor group (performance *M* = 60.56, *SD* = 18.93, *Mdn* = 67.45; *z* = −4.46, *p* < .001, *r* = .83) and the control group (performance *M* = 67.10, *SD* = 8.56, *Mdn* = 69.30; *z* = 4.22, *p* < .001, *r* = .77; see Fig. 1). Both differences represent large effect sizes. The visuomotor group did not differ in performance from the control group (*z* = −.32, *p* = 1.00, *r* = .06).

### Scalp map analyses of EEG power

Next, we investigated the neurophysiological correlates of multi-domain task performance. Kruskal–Wallis tests with the factor group (multi-domain, visuomotor, control) in the three frequency bands for each of the six electrode clusters did not reveal any significant group effects that survived correction for multiple comparisons. We therefore do not report post hoc tests (see Appendix Fig. 4 for a graph about the power values (*Mdn*) for the three groups for each of the six electrode clusters in the three frequency bands).

### Functional brain network analyses

Moving from local scalp map analyses to functional brain networks on the intracranial level, we investigated group differences in functional connectivity with NBS [57] in the three frequency bands theta, alpha1, and alpha2. We hypothesized that the multi-domain group gained most expertise on the multi-domain task as reflected in functional brain network efficiency involved in controlling visuomotor, spatial, and inhibitory functions.

We first tested for a group effect in functional brain networks using an ANOVA with the factor group (multi-domain, visuomotor, control). We only found a main effect of group in the theta band (*t* = 5.6, *p* = .047, FWE-corrected, 49 nodes, 114 edges). Based on this, we tested for the pairwise comparisons of interest, namely (1) the multi-domain group versus the visuomotor group (and vice versa) and (2) the multi-domain group versus the control group (and vice versa). We found a functional brain network significantly differing between the multi-domain and the visuomotor groups, but no functional brain network significantly differing between the multi-domain and the control group. We did not find any significant functional brain networks when testing for the inverse contrasts.

**Fig. 2:**
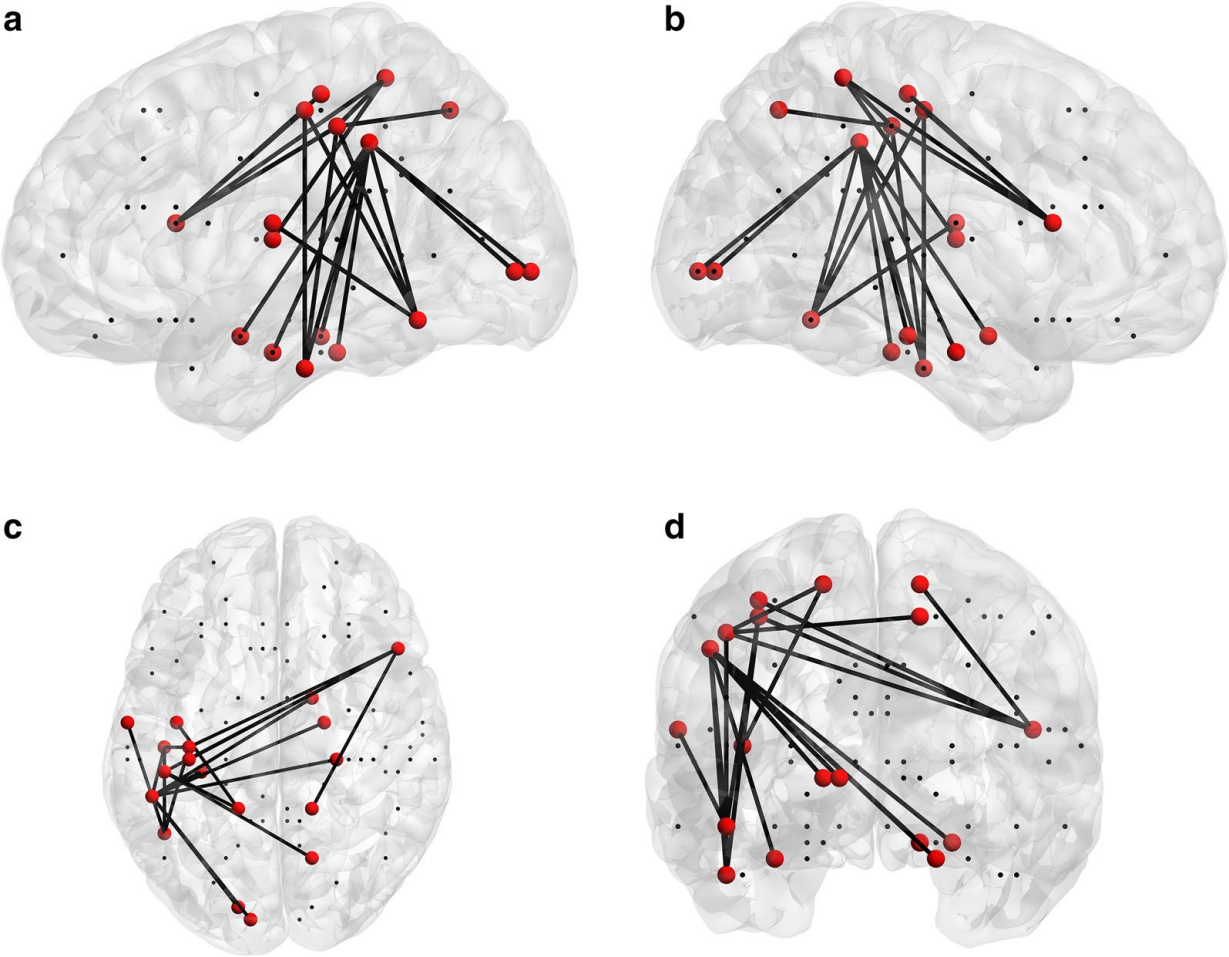
Task-related functional brain network in the theta band. Functional brain network for the comparison of the multi-domain and visuomotor groups during multi-domain task performance. The nodes and edges of the network that survived a threshold of *t* = 3.6 are shown for the sagittal views (**a**, **b**), the axial view (**c**), and the coronal view (**d**). For a specification of the nodes and edges, see Table 3

**Fig. 3:**
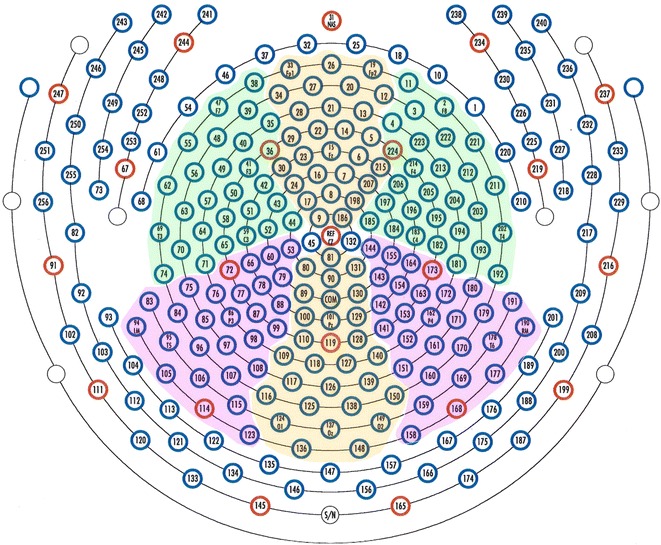
256-Channel map of HydroCel Geodesic Senor Net. *Yellow* anterior middle and posterior middle clusters. *Purple* posterior right and posterior left clusters. *Green* anterior right and anterior left clusters

The multi-domain group showed stronger connectivity as compared to the visuomotor group in a functional brain network encompassing parieto-frontal, parieto-occipital, and parieto-temporal connections (the network consists of 18 nodes and 20 edges, a threshold of *t* = 3.6 was applied, *p* = .006, FWE-corrected; mean connectivity of all edges of the network for the multi-domain group: *M* = .51, *SD* = .14; for the visuomotor group: *M* = .26, *SD* = .11; see Table 3 for the connectivity of each individual edge for each group, see Fig. 2 for a graphical display of the network). This functional brain network was predominantly situated in the left hemisphere with some contralateral connections to the right parahippocampal gyrus and the right inferior frontal gyrus. Overall, the connections corresponded to the task demands: visual and motor areas corresponded to the visual and motor task demands of all three domains, parieto-temporal connections corresponded to the demands of attention, spatial navigation, and memory; and parieto-frontal connections corresponded to demands of inhibition and the simultaneous orchestration of the three tasks. Furthermore, mean connectivity within the functional brain network correlated positively with performance in both groups together (*r*_*s*_ = .52, *p* = .004, *n* = 29; for correlation of single edges with performance, see Table 3). We did not find a significant correlation when correlating performance with mean network connectivity for both groups separately (multi-domain group: *r*_*s*_ = −.20, *p* > .500, *n* = 13; visuomotor group: *r*_*s*_ = −.34, *p* > .200, *n* = 16). For correlations of each edge with performance for both groups together, see Table 3. Correlating performance with connectivity of each edge for each group separately did not reveal any significant correlations.

**Table 3:** Functional brain network in the theta band that significantly differed between the multi-domain and the visuomotor group during multi-domain task performance

Node			Node			Connectivity			
L/R	BA		L/R	BA		*t* value	*r*_1_	*r*_2_	*r*_s_
L	40	Inferior parietal lobule	L	37	Fusiform gyrus, temporo-occipital	4.55	0.61	0.29	.48**
L	40	Inferior parietal lobule	R	28	Parahipppocampal gyrus, temporal	4.01	0.52	0.25	.37
L	40	Inferior parietal lobule	R	34	Parahippocampal gyrus, temporal	3.62	0.52	0.27	.30
L	40	Inferior parietal lobule	R	35	Parahippocampal gyrus, temporal	3.71	0.50	0.25	.35
L	40	Inferior parietal lobule	L	36	Parahippocampal gyrus, temporal	3.65	0.68	0.39	.41*
L	40	Inferior parietal lobule	L	17b	Lingual gyrus, occipital	3.70	0.45	0.21	.42*
L	40	Inferior parietal lobule	L	17a	Lingual gyrus, occipital	3.69	0.42	0.18	.41*
L	40	Inferior parietal lobule	L	20	Fusiform gyrus, temporal	3.65	0.65	0.35	.36
L	2	Postcentral gyrus, primary somatosensory cortex	L	5	Paracentral lobule	4.29	0.67	0.40	.51**
L	2	Postcentral gyrus, primary somatosensory cortex	L	37	Fusiform gyrus, temporo-ocippital	4.14	0.61	0.31	.43*
L	2	Postcentral gyrus, primary somatosensory cortex	L	20	Fusiform gyrus, temporal	3.92	0.69	0.39	.40*
L	2	Postcentral gyrus, primary somatosensory cortex	R	45	Inferior frontal gyrus	4.00	0.17	0.06	.44**
L	2	Postcentral gyrus, primary somatosensory cortex	R	7	Precuneus	3.65	0.49	0.26	.36
R	45	Inferior frontal gyrus	L	4a	Precentral gyrus, primary motor cortex	3.76	0.19	0.05	.59**
R	45	Inferior frontal gyrus	L	4b	Precentral gyrus, primary motor cortex	3.76	0.22	0.06	.54**
R	45	Inferior frontal gyrus	R	5	Paracentral lobule	3.61	0.28	0.09	.42*
L	4b	Precentral gyrus, primary motor cortex	L	20	Fusiform gyrus, temporal	3.74	0.65	0.39	.51**
L	4b	Precentral gyrus, primary motor cortex	L	37	Fusiform gyrus, temporo-occipital	3.68	0.51	0.28	.46*
L	37	Fusiform gyrus, temporo-occipital	L	42	Transverse temporal gyrus	3.73	0.65	0.33	.34
L	5	Paracentral lobule	L	13	Insula	3.65	0.67	0.35	.47*

L, left hemisphere; R, right hemisphere; BA, Brodmann area derived from sLORETA; a, b, refer to different centroid voxels of the same BA (see Table 2); *r*_1_, connectivity of the multi-domain group; *r*_2_, connectivity of the visuomotor group; *r*_s_, the Spearman correlation (for both groups together) of the connectivity of the particular edge with performance. Connectivity values for each edge are based on instantaneous coherence measures. The network consists of 18 nodes and 20 edges. A threshold of *t* = 3.6 was applied

* *p* < .05 uncorrected; ** *p* < .01 uncorrected

#### Regional node analyses

To more closely characterize the functional brain network in the theta band differing between the multi-domain and the visuomotor groups, we calculated the graph-theoretical index weighted node degree that takes into account how strongly each node is connected within the functional brain network. These follow-up analyses are not corrected for multiple comparisons and aim to provide a better picture of the functional brain network revealed by the NBS analyses described above.

First, we averaged the weighted node degree across all nodes of the functional brain network in the theta band. Mean weighted node degree differed significantly between groups (exact Mann–Whitney *U* test: *U* = 154, *p* = .028, *r* = .41). The multi-domain group showed a higher mean weighted node degree (multi-domain group: *M* = 14.09, *SD* = 4.46, *Mdn* = 13.39; visuomotor group: *M* = 10.75, *SD* = 2.86, *Mdn* = 9.99). Furthermore, there were significant group differences in weighted node degree for individual nodes in the lingual gyrus, in the precuneus, and in the paracentral lobule (see Table 4).

**Table 4:** Regional node analyses of the functional brain network revealed by NBS

Node			Weighted node degree				*p* value	*r*
L/R	BA	Brain region	Multi-domain		Visuomotor			
			*M* (*SD*)	*Mdn*	*M* (*SD*)	*Mdn*		
L	17b	Lingual gyrus, occipital	32.00 (5.74)	31.69	26.74 (5.54)	27.22	.022	0.42
L	17a	Lingual gyrus, occipital	29.06 (10.36)	27.61	21.11 (8.66)	20.20	.032	0.40
R	7	Precuneus	17.39 (8.03)	17.77	11.66 (4.13)	10.36	.015	0.45
R	5	Paracentral lobule	16.21 (8.66)	16.73	9.29 (4.00)	8.37	.015	0.45

Nodes of the network in the theta band that differ in weighted node degree between the multi-domain group and the visuomotor group are displayed

L, left hemisphere; R, right hemisphere; BA, Brodmann area derived from sLORETA; *M*, mean; *SD*, standard deviation; *r*, effect size. The weighted node degree was compared between groups with the Mann–Whitney *U* test. Exact *p* values are reported uncorrected

Mean weighted node degree of the functional brain network in the theta band did not correlate with performance across both groups, nor were there correlations within each group separately. With regard to individual nodes, weighted node degree in a node of the primary motor cortex (BA 4a; *r*_*s*_ = .37, *p* = .048, *n* = 29) correlated positively with performance across both groups. However, excluding one participant of the multi-domain group showing a weighted node degree value beyond four standard deviations reduced this correlation (BA 4a; *r*_*s*_ = .32, *p* = .093, *n* = 28). Other weighted node degree values of individual nodes did not show any correlations with performance, neither across both groups nor within each group separately.

## Discussion

The principal finding of the present study is that training-related expertise was reflected in differences in behavioral performance and functional brain network connectivity one year after training. Based on their specific training history, the participants of the multi-domain group had expertise in handling an inhibition, a visuomotor, and a spatial navigation task simultaneously, while the participants of the visuomotor group only had expertise in visuomotor tasks. In line with this, the multi-domain group performed the multi-domain task significantly better than the visuomotor group and the control group. However, the expertise of the visuomotor group did not benefit performance on the multi-domain task. Better performance of the multi-domain group was paralleled by stronger theta connectivity in a functional brain network subserving task performance. This network encompassed visual, motor, executive, and memory-associated brain areas. Connections to visual and motor areas corresponded to the visual and motor task demands of all three domains, parieto-temporal connections corresponded to the demands of attention, spatial navigation, and memory, and parieto-frontal connections corresponded to demands of inhibition and the simultaneous orchestration of the three tasks. Mean connectivity of the functional brain network in the theta band of both groups correlated positively with performance. With regard to weighted node degree, indicating the importance of nodes in a network, the multi-domain group showed higher values in areas important for visual processing (BA 17), somatosensory processing (BA 5), and the precuneus (BA 7) for visuospatial processing and memory [62]. Taken together, the multi-domain group showed more efficient information transmission as indicated by stronger connections and higher mean weighted node degree.

Aging is generally associated with changes in network characteristics that move away from optimal small-world networks [16–19]. Interestingly, the present study found that older adults who acquired an expertise through intensive multi-domain training show more efficient information processing within a task-related functional brain network in the theta frequency band one year after training as compared to those originally trained in visuomotor function. This task-related functional brain network encompassed brain regions that have shown the most prominent changes with aging, such as prefrontal and temporal regions including the hippocampus [3]. However, as our analyses is only cross-sectional, we cannot draw conclusions about the healthy aging process and how these functional brain network changes might be associated with it.

It remains a matter of investigation how specific such changes in functional brain network characteristics are. With respect to the specificity of the frequency bands, we did not find a main effect of group for the two alpha frequency bands. Theta frequencies have been associated with cognitive control processes [28–30] and working memory training has shown to increase small-world topology in the theta frequency band in young adults [26]. Cognitive control was also required for the multi-domain task of the present study. However, we do not have a clear explanation why we did not find any group effects in the alpha frequency bands.

Furthermore, do groups differ with respect to their functional brain network characteristics only during performance on the particular training task or are there task-independent functional brain network changes? Experience-dependent alterations have been shown in resting state [63] as well as in task-related functional brain networks [34, 35, 37]. With regard to resting state, professional musicians showed increased functional connectivity in motor, visual, auditory, and somatosensory cortices [63]. With regard to task-related functional connectivity patterns, professional racing-car drivers showed enhanced functional connectivity in task-relevant brain areas during a motor reaction and a visuospatial task when compared to naive drivers [35]. This could be seen as a transfer effect since they had never performed on the motor and visuospatial task before. It is likely the proficiency in racing-car driving to which differences in functional brain networks important for performance on these tasks can be attributed. Thus, there is evidence for brain network changes under different conditions, such as during resting state, during task performance, and during performance on transfer tasks.

The relationship between expertise and performance is not linear in our findings. We found that the multi-domain group with the most expertise on the multi-domain task outperformed the visuomotor group and the control group. The visuomotor group and the control group did not differ in performance, which does not support the assumption that prior visuomotor training potentially benefits the visuomotor domain of the multi-domain task. Moving to functional brain networks, we found a functional brain network differing between the multi-domain group and the visuomotor group that paralleled the performance difference. In contrast, there were no significant functional brain network differences between the control group and the multi-domain group although the multi-domain group showed significantly better performance. Comparing the multi-domain group with the control group, it has to be taken into account that they differed with respect to past training experience. Hence, a different mechanism might account for the performance difference between the multi-domain group and the control group. One could speculate that the control group activated a similar functional brain network, but this was inefficient since performance was significantly worse. Another possible explanation of the result pattern is that functional brain networks were impaired in the visuomotor group because they had a training history that was contextually similar, but different with respect to the functions targeted by the assessed multi-domain task, and that this impaired their functional brain networks more strongly than those of the naive controls.

### Limitations

The present study is based on a cross-sectional comparison of three different groups differing with regard to their cognitive and motor training histories. The number of participants in each group is rather small, which limits statistical power. Furthermore, to gain a better understanding of how training beneficially affects functional brain networks, longitudinal studies are necessary for insights into training-related change [see e.g., 26, for working memory training in young adults]. Technical limitations of the iPad-based Hotel Plastisse multi-domain task did not allow sending of task-related triggers to the EEG system. Due to the naturalistic approach of the iPad game, participants were generally engaged in the task and used different strategies to handle the complex multi-domain tasks challenging inhibiton, visuomotor function, and spatial navigation. Furthermore, we fixed task difficulty of the multi-domain task to an intermediate level. As the multi-domain training group trained on this task during their prior intensive training period, the task is likely to have been easier for this group than for the visuomotor group and the control group. However, adapting the task demands for each group (i.e., by choosing different difficulty levels) would induce other confounds due to dissimilar tasks in EEG (higher difficulty levels would, for example, be associated with longer labyrinths and shorter delays between the go/no-go stimuli). Inducing different task demands is a general problem when comparing experts and novices. However, a measure of (subjective) task demand and effort should be included in future studies. Despite these limitations, we believe that the current study uses an innovative training task as a first step to investigate expertise-related differences in cognitive and motor performance during healthy aging.

### Conclusion and implications

Cross-sectional studies comparing groups with different expertise levels have revealed that expertise is associated with beneficial functional brain network characteristics [25, 34, 35, 37]. In line with these findings, two of the three groups examined in our study had developed a particular expertise due to their specific training histories. Depending on that expertise, participants differed in their functional brain network characteristics during task performance. The multi-domain group showed higher functional brain network efficiency even one year after training. Such group differences in functional brain network characteristics within a sample of healthy older adults point to the potential to modify typical age-related changes, tending towards less optimal brain network characteristics. Longitudinal studies will be important to characterize brain network changes more specifically over time and identify factors that are associated with brain network changes during the aging process. A better understanding of the neurobiological bases of aging and plasticity would provide valuable information concerning beneficial activities and lifestyle factors that can counter age-related cognitive decline and preserve cognitive functioning. Insights into beneficial (and detrimental) factors can eventually lead to recommendations for older adults who want to preserve their cognitive and neural functioning into older ages.

## Figures and Tables

**Fig. 4:**
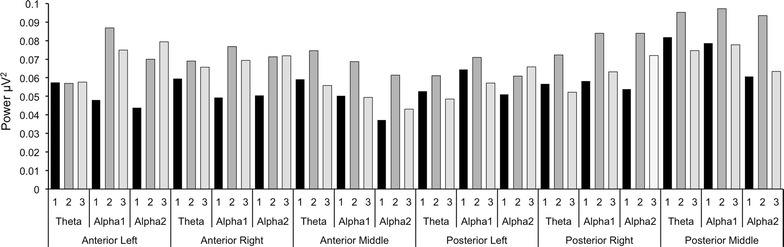
EEG power. Power values (*Mdn*) for the three groups (1 = multi-domain group, 2 = visuomotor group, 3 = control group) for each of the six clusters and the frequency bands theta, alpha1, and alpha2. See Appendix Fig. 3 and Table 5 for information on how the clusters were defined

## Appendix

See Table 5 and Figs. 3 and 4.

**Table 5:** Electrode numbers for each cluster

Electrode cluster	Number of electrodes
Anterior left	E44; E43; E52; E42; E51; E59; E36; E41; E50; E58; E65; E35; E40; E49; E57; E64; E71; E39; E48; E56; E63; E70; E38; E47; E55; E62; E69; E74
Anterior middle	E9; E186; E17; E8; E198; E24; E16; E7; E207; E30; E23; E15; E6; E215; E29; E22; E14; E5; E28; E21; E13; E34; E27; E20; E12; E33; E26; E19
Anterior right	E185; E184; E197; E196; E183; E206; E182; E195; E205; E214; E224; E181; E194; E204; E213; E223; E4; E193; E203; E212; E222; E3; E192; E202; E211; E221; E2; E11
Posterior left	E53; E60; E79; E66; E78; E88; E72; E77; E87; E99; E76; E86; E98; E75; E85; E97; E108; E84; E96; E107; E83; E95; E106; E115; E94; E105; E114; E123
Posterior middle	E81; E80; E90; E130; E131; E89; E100; E101; E129; E128; E119; E110; E109; E118; E127; E140; E117; E126; E139; E116; E125; E138; E150; E124; E137; E149; E136; E148
Posterior right	E144; E143; E155; E164; E154; E142; E173; E163; E153; E141; E172; E162; E152; E180; E171; E161; E151; E179; E170; E160; E191; E178; E169; E159; E190; E177; E168; E158
